# Heterogeneity of Prevalence of Social Media Addiction Across Multiple Classification Schemes: Latent Profile Analysis

**DOI:** 10.2196/27000

**Published:** 2022-01-10

**Authors:** Cecilia Cheng, Omid V Ebrahimi, Jeremy W Luk

**Affiliations:** 1 Department of Psychology The University of Hong Kong Pokfulam Hong Kong; 2 Department of Psychology University of Oslo Oslo Norway; 3 Research Institute Modum Bad Psychiatric Hospital Vikersund Norway; 4 National Institute on Alcohol Abuse and Alcoholism Bethesda, MD United States

**Keywords:** behavioral addiction, compulsive social media use, information technology addiction, mental health, psychological assessment, sensitivity, social network site, social networking, well-being

## Abstract

**Background:**

As social media is a major channel of interpersonal communication in the digital age, social media addiction has emerged as a novel mental health issue that has raised considerable concerns among researchers, health professionals, policy makers, mass media, and the general public.

**Objective:**

The aim of this study is to examine the prevalence of social media addiction derived from 4 major classification schemes (strict monothetic, strict polythetic, monothetic, and polythetic), with latent profiles embedded in the empirical data adopted as the benchmark for comparison. The extent of matching between the classification of each scheme and the actual data pattern was evaluated using sensitivity and specificity analyses. The associations between social media addiction and 2 comorbid mental health conditions—depression and anxiety—were investigated.

**Methods:**

A cross-sectional web-based survey was conducted, and the replicability of findings was assessed in 2 independent samples comprising 573 adults from the United Kingdom (261/573, 45.6% men; mean age 43.62 years, SD 12.24 years) and 474 adults from the United States (224/474, 47.4% men; mean age 44.67 years, SD 12.99 years). The demographic characteristics of both samples were similar to those of their respective populations.

**Results:**

The prevalence estimates of social media addiction varied across the classification schemes, ranging from 1% to 15% for the UK sample and 0% to 11% for the US sample. The latent profile analysis identified 3 latent groups for both samples: low-risk, at-risk, and high-risk. The sensitivity, specificity, and negative predictive values were high (83%-100%) for all classification schemes, except for the relatively lower sensitivity (73%-74%) for the polythetic scheme. However, the polythetic scheme had high positive predictive values (88%-94%), whereas such values were low (2%-43%) for the other 3 classification schemes. The group membership yielded by the polythetic scheme was largely consistent (95%-96%) with that of the benchmark.

**Conclusions:**

Among the classification schemes, the polythetic scheme is more well-balanced across all 4 indices.

## Introduction

### Background Context

The internet has turned the world into an interconnected, global village in which information and problems alike spread swiftly across countries. Apart from face-to-face interactions, social media has emerged as a major channel of interpersonal communication in the cyber age. Social media use is beneficial in many aspects. For instance, social media allows people to maintain contact with existing friends or family members who live apart [1,2], and individuals who use social media to connect with their social network members tend to experience greater levels of subjective well-being [3,4]. In the extended period of social distancing during the COVID-19 pandemic, social media use is found to be associated with both social and physical well-being [5,6]. For people who seek mental health professional services, the interventions delivered through social media are deemed more accessible and engaging than face-to-face interventions [7].

Despite these psychological benefits, the misuse of social media can incur considerable psychological costs, especially for individuals who use social media as a refuge to evade unpleasant feelings or real-life problems. According to the model of compensatory internet use [8], social media use may improve these individuals’ psychological condition in the short run, but such short-term benefits may also strengthen their dependence on social media, leading to continuous excessive use of social media and the development of social media addiction [9]. Moreover, social media addiction is motivated by the need to fulfill fundamental psychological needs that cannot be gratified in the real world, such as the need to belong [10-13].

### Social Media Addiction and Its Assessment

Social media addiction is a type of behavioral addiction that is broadly defined as compulsive engagement in social media platforms that significantly disrupts the users’ functioning in important life domains, such as interpersonal relations, work or study performance, and physical health [14,15]. According to the components model of behavioral addiction [16], social media addiction is conceptualized as a set of symptoms pertaining to six types of problematic behavior: (1) salience, which refers to the dominance of social media activities in one’s thoughts and daily life; (2) tolerance, which refers to the tendency of spending an increasing amount of time using social media to attain the same amount of pleasure; (3) mood modification, which refers to the use of social media to avoid or mitigate unpleasant emotions experienced in real-life events; (4) relapse, which refers to the failure of curbing excessive social media use after attempts of abstinence or control; (5) withdrawal, which refers to psychological distress experienced when one cannot get access to social media; and (6) conflict, which refers to the adverse impact on one’s job or studies because of problematic social media use.

Although social media addiction is not currently a diagnosable condition, researchers have constructed measures of social media addiction based on the diagnostic criteria for other behavioral addictions such as gambling disorder [16,17]. The most popular ones include the Facebook Intrusion Questionnaire [18] and the Bergen Social Media Addiction Scale (BSMAS) [19]. The former measure assesses addiction specific to a single social media platform, whereas the latter assesses addiction to social media in general. Instead of creating new assessment tools, another group of researchers have modified the current validated scales of internet addiction. The modification typically involves altering the context from *Internet* to *Facebook* or *social media*. For instance, the items of the Problematic Facebook Use Scale [20] were adapted from those of the Generalized Problematic Internet Use Scale [21].

### Classification Schemes for Social Media Addiction

Validated measures of social media addiction have been widely used as screening tools for distinguishing individuals with and without the problem [22,23]. Both monothetic and polythetic formats have been adopted to yield prevalence estimates and to screen cases [24,25], because these schemes have long been used in case classifications of psychiatric disorders in the Diagnostic and Statistical Manual of Mental Disorders (DSM) [17,26,27]. The *classical* monothetic classification is generally regarded as more conservative because a positive diagnosis requires the endorsement of all the listed criteria [28]. For polythetic classification, however, no single criterion is required to make a diagnosis. The polythetic classification is more liberal than the monothetic classification because a positive diagnosis requires the endorsement of more than half of the listed criteria rather than all; therefore, individuals with the same classification may have different clinical presentations. Polythetic classification is commonly used in a variety of clinical diagnoses, including gambling disorder and substance abuse [17,27].

Most existing measures of social media addiction consist of items that are answered on a Likert-type scale ranging from 1 to 5 rather than dichotomous options. A usual practice for indicating the presence of a symptom involves the recoding of the 5-point ratings using the midpoint (ie, 3) as the cutoff such that a particular criterion is met for a score of 3 or above. Some researchers recently advocated stricter coding by setting a higher cutoff of 4 instead of the midpoint [29,30]. Taken together, a review of the literature reveals 4 commonly adopted classification schemes: monothetic, polythetic, strict monothetic, and strict polythetic. As these schemes vary in the extent of strictness in case classifications, different prevalence estimates are obtained, with higher prevalence yielded from more liberal classifications such as the polythetic scheme.

Previous studies on social media addiction have adopted either 1 or at most 2 of the classification schemes for deriving prevalence estimates, and the reported prevalence rates differ vastly across studies. As social media addiction is a global mental health concern, researchers worldwide have investigated the prevalence of this emergent problem [31-34]. The samples recruited in these prevalence studies vary considerably in their demographic characteristics, such as age and ethnicity, making between-study comparisons difficult. This study is the first to apply all 4 major classification schemes such that comparisons of the prevalence drawn from various schemes can be made. A total of 2 independent, demographically heterogeneous samples were included to evaluate the extent of cross-sample replicability in the findings.

### Evaluation of Classification Schemes for Social Media Addiction

**Figure 1:**
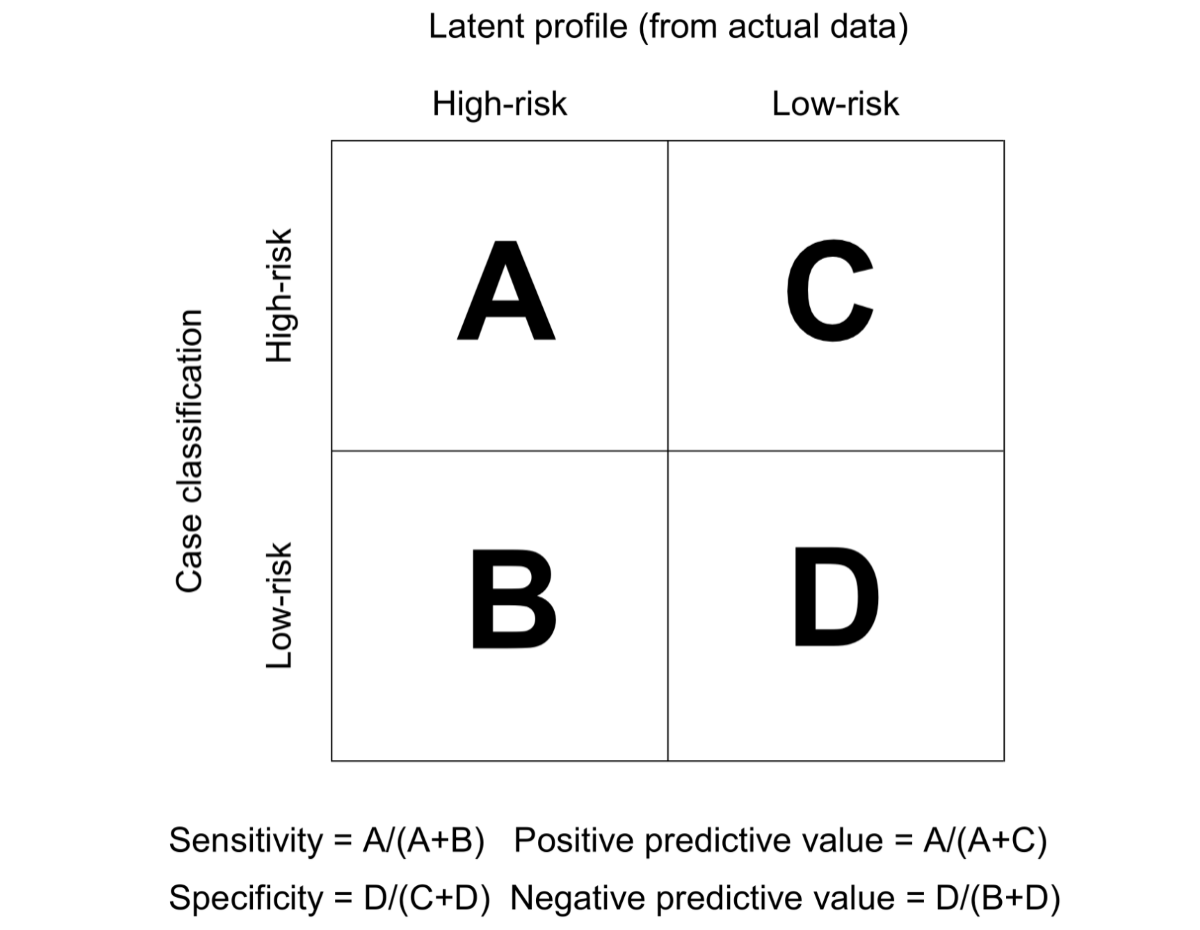
The 4 indicators of sensitivity and specificity analysis.

Sensitivity and specificity analyses are widely used for the evaluation of classification schemes [35]. This method seeks to test the performance of a classification in matching and predicting a diagnosis or outcome. As a *gold standard* for classifying social media addiction is currently unavailable, the latent profiles embedded in a data set are adopted as a benchmark to evaluate which existing classification scheme for social media addiction can provide the best fit to empirical data. Latent profile analysis (LPA) is a type of mixture modeling that can reveal hidden subgroups of individuals from observed data that share similar symptom profiles [36]. This person-centered statistical approach is especially appropriate for classifying disorders with heterogeneous symptoms, because highly consistent classifications can be obtained by precise distinctions among profiles and differences in characteristics among profiles [37,38]. LPA has been applied to social media addiction and can be used to estimate the proportion of the population with different risk levels of this disorder [39]. This study extends the literature by evaluating the extent to which the prevalence estimate derived from a particular classification approximates the latent profiles of symptoms actually endorsed by respondents, and the results are indicated by 4 indices: sensitivity, specificity, positive predictive value, and negative predictive value (Figure 1).

In this study, sensitivity refers to the proportion of individuals from the data-driven, latent high-risk group who are also classified by a particular scheme as high risk. Specificity refers to the proportion of individuals from the data-driven, latent low-risk group who are also classified by a particular scheme as low risk. Positive predictive value refers to the proportion of high-risk participants classified by a particular scheme who also belong to the data-driven, latent high-risk group. Negative predictive value refers to the proportion of low-risk participants classified by a particular scheme who also belong to the data-driven, latent low-risk group.

### Social Media Addiction and Comorbid Mental Health Problems

To establish that social media addiction is not merely a normative behavioral pattern, it is important to evaluate whether it is associated with other comorbid mental health problems. In the literature on social media addiction, a frequently researched psychiatric condition is depression, which has been found to have positive associations with social media addiction. Specifically, individuals with depression tend to have a high risk of developing social media addiction [40,41]. Although users generally have more pleasant experiences than unpleasant ones when engaging in social media, unpleasant experiences on social media (eg, cyberbullying and social comparison with friends or strangers) tend to compromise mental health [42]. These adverse mental health impacts are especially salient among individuals with depression owing to their ruminative response style [43,44].

Another frequently researched psychiatric condition is anxiety [10,45]. Some studies have shown that individuals with anxiety are prone to social media addiction because of their strong motivation to avoid face-to-face social interactions [46]. As individuals with social media addiction tend to spend more time on web-based than in-person social interactions, their prolonged social media use may erode social skills and promote greater fear of meeting people in real life [47]. Their anxiety may in turn aggravate their symptoms of social media addiction, as these individuals continue to perceive in-person interactions as a source of threat [46]. People who fear face-to-face communication experience a deficit in need for relatedness, which may arise from feelings of insecurity in daily life situations, and social media may thus be used as a compensational tool to gratify their relatedness needs [47]. Prolonged, excessive use of social media may lead to social media addiction among individuals with heightened anxiety [48].

## Methods

### Participant Recruitment and Procedures

The 2 independent samples for this study were recruited from Prolific because this web-based participant pool provides heterogeneous samples with diverse demographic characteristics. Moreover, the participants of this web-based survey platform were found to be more honest and naïve, and their data quality was better than that of members from other survey platforms [49]. Eligible participants were residents of the United Kingdom or the United States aged between 18 and 65 years and were users of at least one social networking site. To maintain data quality, only those who had an approval rating of ≥90% on the survey site were included.

The samples were recruited from the United Kingdom and the United States because members of both countries are the largest body of consumers of the English version of the BSMAS. These countries are appropriate for sample replication because they are highly similar in their internet penetration rates, socioeconomic development, and cultural values [50,51]. Recruitment was carried out from May 18, 2020, to May 24, 2020.

All participants completed a set of questionnaires that was constructed and launched through a web-based survey tool, Qualtrics (Qualtrics International Incorporation). The research protocol of this study followed those adopted in previous web-based surveys [29,52]. An advertisement was placed on the survey platform, and those interested were invited to sign up. All participants had to give their consent before the survey began. Upon completion of the survey, they were paid according to the standard rate set by the survey platform. The human research ethics committee of the university where the principal author is employed approved the research protocol before this study was implemented.

### Measures

#### Social Media Addiction

The symptoms of social media addiction were measured by the BSMAS [10], which is by far the most widely used validated assessment tool for addictive use of social media in general. This scale comprises 6 items, each measuring a core symptom (ie, salience, tolerance, mood modification, loss of control, withdrawal, and conflict). Item wording was consistent with the diagnostic criteria for gambling disorder [17]. The respondents rated each item on a 5-point scale, ranging from 1 (*very rarely*) to 5 (*very often*). The item scores were aggregated to obtain a composite score, and the 4 major classification schemes outlined in the *Introduction* section (the *Classification Schemes for Social Media Addiction* subsection) were adopted to categorize respondents into groups: high- versus no- to-low-risk. Prevalence rate refers to the proportion of participants who were classified as having social media addiction to the entire sample, and the prevalence rates derived from various classification schemes were then compared. The BSMAS was a reliable measure in this study (Cronbach α=.88 and .86 for the UK and the US samples, respectively).

#### Depression

In this study, depression and anxiety were included as criterion variables owing to the high comorbidity between social media addiction and both of these mental health conditions [53,54]. Probable depression was assessed using the Center for Epidemiological Studies Depression Scale [55], which was constructed for use with general, nonpsychiatric populations. Respondents were instructed to rate each of the 20 items on a 4-point scale ranging from 0 (*rarely or none of the time*) to 3 (*most or all of the time*). A higher composite score indicated a higher level of depression. This measure is widely adopted as a screening tool for clinical depression, with a recommended cutoff score of 16. This threshold score was thus adopted to indicate probable depression. The measure exhibited excellent psychometric properties in screening for major depression in the general population as verified by the DSM [55]. The depression measure displayed internal consistency in this study (Cronbach α=.92 and .91 for the UK and the US samples, respectively).

#### Anxiety

Probable anxiety was measured by the state anxiety subscale of the State-Trait Anxiety Inventory Form Y1 [56], which was selected because this extensively validated measure has by far been the most popular screening tool for anxiety [57]. Respondents rated each of the 20 items on a 4-point scale ranging from 1 (*not at all*) to 4 (*very much so*). A higher composite score indicates a higher level of anxiety. According to the community adult norms stated in the manual [56], a cutoff score of 40 was used for screening. This measure was found to be reliable (Cronbach α=.96 and .96 for the UK and the US samples, respectively).

### Data Analysis

LPA was conducted because this person-centered approach is currently widely applied for identifying latent groups with similar characteristics [58]. A total of 6 BSMAS items were included as indicator variables in this analysis. Multiple indices were checked to determine the model with the best fit of data [59]. Specifically, a number of models with different class solutions (*k* ranging from 2 to 5) were tested, and better data fit was indicated by lower values of 3 goodness-of-fit statistics: Bayesian Information Criterion, sample size adjusted Bayesian Information Criterion, and Akaike Information Criterion. Entropy was also examined to evaluate the precision of assigning latent group membership, with a value of ≥0.80, indicating precision of classification. A class solution with an entropy value of <0.80 was ruled out. In addition, the Lo-Mendell-Rubin likelihood ratio test and bootstrap likelihood ratio test were used to assist model selection, and a significant result (ie, *P*<.05) showed that a *k* class model improved a *k*–1 class model. If the results revealed that 2 or more models were adequate, model parsimony and interpretability were considered. After the model selection decision had been made, the profiles were plotted for each group using a line graph.

The latent profiles identified in the LPA were then mapped onto existing classification schemes using sensitivity and specificity analyses [60]. A total of 4 indices—sensitivity, specificity, positive predictive value, and negative predictive value—were examined (the *Evaluation of Classification Schemes for Social Media Addiction* subsection and Figure 1). In addition, an index of overall consistency was reported to indicate the percentage of overlap in group membership between the latent profiles and high-versus-low-risk groups classified by a particular scheme.

Risk ratio was used to make two types of estimation: (1) the individual contribution of demographic variables (sex and age groups) to the risk for social media addiction and (2) the individual contribution of social media addiction prevalence to the risk for mental health problems. All statistical analyses were conducted using SPSS version 26 (IBM), except for LPA, which was conducted using MPlus version 8.5 (Muthén and Muthén).

### Funding and Ethical Considerations

This study was funded in part by the General Research Fund administered by the Research Grants Council of Hong Kong in January 2020 (grant 17400714). The research protocol was reviewed and approved by the human research ethics committee of the University of Hong Kong before data collection (approval number: EA2002033**;** approval date: March 4, 2020). All study procedures were performed in accordance with the ethical principles of the Declaration of Helsinki. All participants were required to provide informed consent before completing the survey.

## Results

### Sample Characteristics

The UK sample comprised 573 adults, whereas the US sample comprised 474 adults. The sample size of each country met the requirements for conducting covariance modeling [59] and sensitivity and specificity analyses [35]. The average age of the participants in the UK sample was 43.62 years (SD 12.24 years) and that of the participants in the US sample was 44.67 years (SD 12.99 years). Table 1 presents the sex and age distribution of the participants from both samples as well as those of the UK and the US populations. As shown in Table 1, sample distributions of these major demographic variables were comparable to those of their respective populations.

**Table 1:** Sex and age distribution of the 2 samples compared with that of their own population (N=1047).

Parameters		The United Kingdom, n (%)		The United States, n (%)	
		Sample (n=573)	Population in 2020	Sample (n=474)	Population in 2020
**Sex**					
	Male	261 (45.6)	33,821^a^ (49.8)	224 (47.4)	165,899^a^ (50.1)
	Female	312 (54.4)	34,065 (50.2)	250 (52.6)	165,104 (49.9)
**Age group (years)**					
	18-34	192 (33.5)	22,775 (33.5)	169 (35.6)	118,036 (35.6)
	35-49	191 (33.3)	22,654 (33.4)	152 (32.1)	105,788 (32)
	50-65	190 (33.2)	22,457 (33.1)	153 (32.3)	107,179 (32.4)

^a^Figures in these columns are expressed in thousands.

### Case Classification With LPA

Table 2 presents the results of LPA that were tested and compared among the 4 models derived from different classification schemes. For the UK sample, the 3-class model was selected because the Lo-Mendell-Rubin likelihood ratio test showed that this model had better data fit than the 2-class model, but the degree of data fit was highly similar for the 3-class, 4-class, and 5-class models. Hence, the 3-class model was chosen for parsimonious considerations.

**Table 2:** Summary of latent profile analysis comparing the various models (N=1047).

Characteristics		Model comparison			
		2-class model	3-class model	4-class model	5-class model
**The UK Sample (n=573)**					
	BIC^a^	8748.65	8196.68	8063.43	7882.23
	SSABIC^b^	8688.33	8114.14	7958.67	7755.25
	AIC^c^	8665.99	8083.56	7919.85	7708.20
	Entropy	0.94	0.90	0 90	0.93
	LMR-LRT^d^, *P* value	.004	.001	.32	.08
	BLRT^e^, *P* value	<.001	<.001	<.001	<.001
**The US Sample (n=474)**					
	BIC	6755.99	6342.76	5946.03	5855.20
	SSABIC	6695.69	6260.24	5841.29	5728.24
	AIC	6676.93	6234.56	5808.71	5688.75
	Entropy	0.93	0.91	0.93	0.93
	LMR-LRT, *P* value	<.001	.02	.005	.09
	BLRT, *P* value	<.001	<.001	<.001	<.001

^a^BIC: Bayesian Information Criteria.

^b^SSABIC: sample size adjusted Bayesian Information Criterion.

^c^AIC: Akaike Information Criterion.

^d^LMR-LRT: Lo-Mendell-Rubin likelihood ratio test.

^e^BLRT: bootstrap likelihood ratio test.

**Figure 2:**
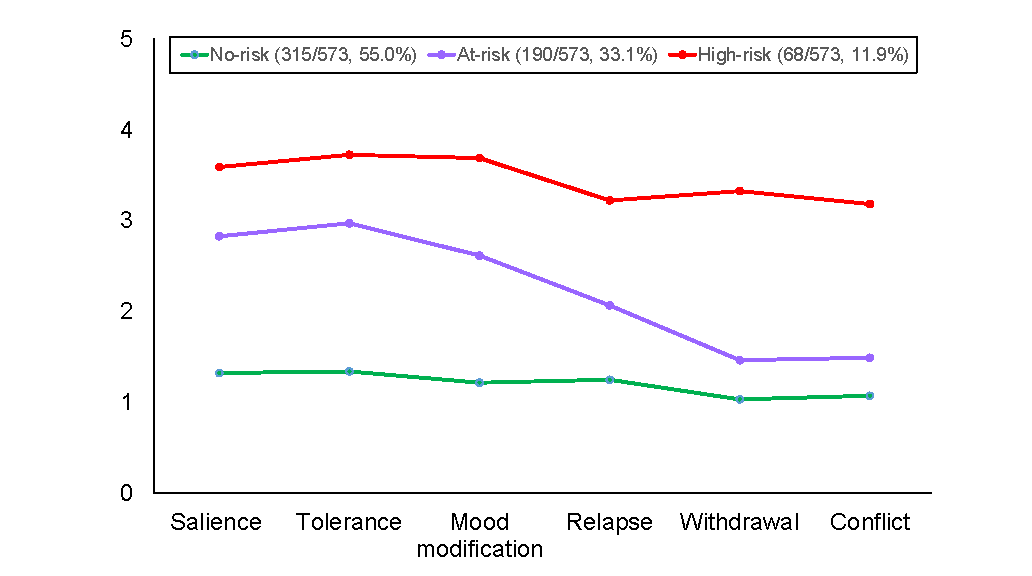
Latent profiles for the 3-class solution for the UK samples.

The latent profiles of the participants from the United Kingdom are shown in Figure 2. As shown in this figure, the 3 latent groups differed in terms of symptom severity. Specifically, more than half of the participants were assigned to the first group (315/573, 55%) characterized by low mean item scores across all 6 criteria of social media addiction (<1.34), and this group was labeled as *low-risk*. The profile pattern of the second group (190/573, 33.1%) was relatively more complex than that of the first group. The participants from the second group were more likely to endorse the salience, tolerance, and mood modification criteria, with item mean scores clustered around the midpoint (range 2.62-2.97), but not the remaining 3 criteria (mean item scores<2.07). The second group was labeled as *at-risk* because the mean item scores for half of the criteria approached the cutoff (ie, midpoint) for both the monothetic and polythetic schemes. The third group (68/573, 11.9%) had high mean item scores for all the criteria that were above the cutoff for the monothetic and polythetic schemes (>3.18), and this group was labeled as *high-risk*.

**Figure 3:**
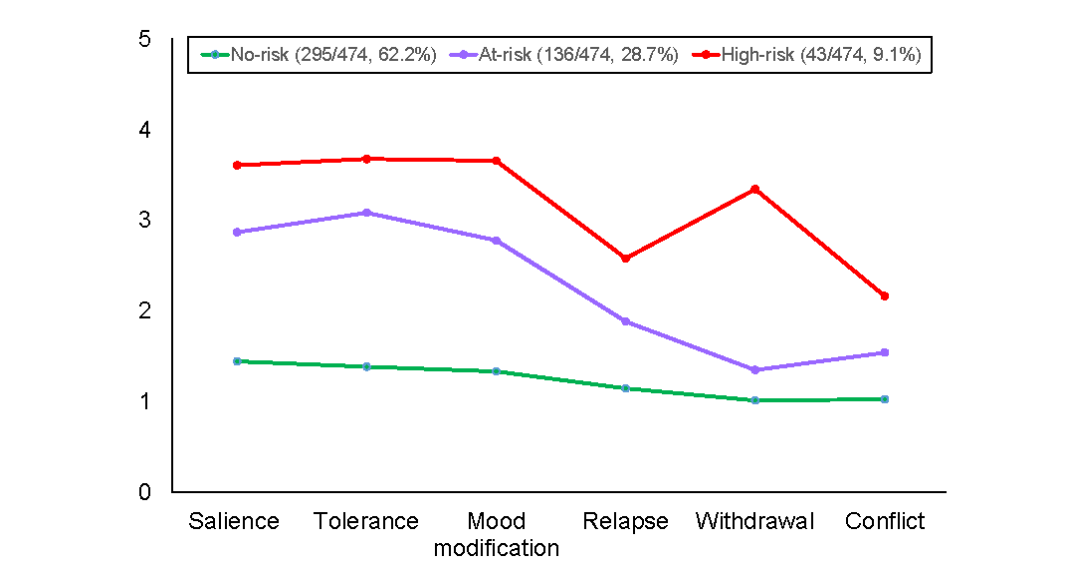
Latent profiles for the 3-class solution for the US samples.

For the US sample, both the 3-class and the 4-class models demonstrated good data fit, but the former model was chosen because its grouping of participants was more interpretable than that of the latter. The 3 latent groups are shown in Figure 3. As revealed in this figure, the profiles of the first 2 groups were highly similar for the UK and the US samples. Specifically, the low-risk group of the US sample (295/474, 62.2%) had low mean item scores across all 6 criteria (<1.45), and the at-risk group (136/474, 28.7%) tended to endorse the same 3 criteria (salience, tolerance, and mood modification; mean item scores ranged from 2.78 to 3.08) but not the other 3 (mean item scores<1.89).

Although the profiles of the low-risk and at-risk groups of participants from the United States were comparable with those of the same 2 groups of the participants from the United Kingdom, the profiles of the third group were different for the 2 samples. Instead of consistently having all scores above the midpoint across all 6 items as their UK counterparts did, the third group of the US sample (43/474, 9.1%) had mean item scores above the midpoint only for two-thirds of the criteria (ie, salience, tolerance, mood modification, and withdrawal; mean item scores>3.34). The third group of the US sample was also labeled as *high-risk* owing to their members’ endorsement of 4 out of 6 criteria that was consistent with the polythetic scheme.

Ad hoc Bonferroni tests were conducted among the 3 latent groups for each of the 6 criteria. The results consistently showed that all the criterion scores of the high-risk group were significantly higher than those of the at-risk group, whose criterion scores were in turn significantly higher than those of the low-risk group (*P*<.001). These results provided evidence for the distinctness of the profiles of the 3 latent groups.

### Prevalence of Social Media Addiction

As both of the samples were heterogeneous in terms of demographic characteristics, risk ratios were computed to identify sex and age differences. The results revealed considerable age differences in the prevalence of social media addiction for both samples (Table 3). For the UK sample, younger adults aged between 18 and 34 years were at a 4-fold higher risk of social media addiction than their older counterparts aged between 50 and 65 years, and this pattern of findings was consistent across the various classification schemes. For the US sample, the risk of social media addiction was 2 to 4 times higher in the group of younger adults compared with the group of older adults. However, sex differences in the prevalence of social media addiction were not prominent for both samples, except female participants from the US sample were found to have higher risks of social media addiction than their male counterparts when the strict polythetic scheme was adopted for classification.

**Table 3:** Prevalence of social media addiction by major classification scheme, sex, and age group for the 2 samples (N=1047).

Parameters				Classification scheme																		
				Strict monothetic, n (%)		Strict monothetic, RR^a^		Strict polythetic, n (%)		Strict polythetic, RR		Monothetic, n (%)		Monothetic, RR		Polythetic, n (%)		Polythetic, RR		Latent profile^b^, n (%)		Latent profile^b^, RR
**The UK Sample (n=573)**																						
	Total		4 (0.7)		—^c^		23 (4)		—		29 (5.1)		—		86 (15)		—		68 (11.9)		—	
	**Sex**																					
		Male	7 (1.2)		N/A^d^		28 (4.9)		1.56		35 (6.1)		1.58		101 (17.6)		1.53		72 (12.6)		1.18	
		Female	0 (0)		Ref^e^		18 (3.1)		Ref		22 (3.8)		Ref		66 (11.5)		Ref		62 (10.8)		Ref	
	**Age group (years)**																					
		18-34	12 (2.1)		4.03		48 (8.4)		4.09		60 (10.5)		4.09		144 (25.1)		3.78		132 (23)		6.20	
		35-49	0 (0)		N/A		17 (3)		1.45		22 (3.8)		1.49		90 (15.7)		2.36		63 (11)		2.41	
		50-65	3 (0.5)		Ref		12 (2.1)		Ref		15 (2.6)		Ref		38 (6.6)		Ref		26 (5)		Ref	
**The US sample (n=474)**																						
	Total		1 (0.2)		—		12 (2.5)		—		12 (2.5)		—		52 (11)		—		43 (9.1)		—	
	**Sex**																					
		Male	2 (0.4)		N/A		21 (4.4)		4.38		13 (2.7)		1.23		64 (13.5)		1.66		47 (9.9)		1.25	
		Female	0 (0)		Ref		4 (0.8)		Ref		11 (2.3)		Ref		39 (8.2)		Ref		39 (8)		Ref	
	**Age group (years)**																					
		18-34	0 (0)		N/A		16 (3.4)		2.24		20 (4.2)		4.19		72 (15.2)		2.16		60 (12.7)		2.74	
		35-49	3 (0.6)		N/A		15 (3.2)		2.09		15 (3.2)		3.13		60 (12.7)		1.79		54 (11.4)		1.13	
		50-65	0 (0)		Ref		7 (1.5)		Ref		5 (1.1)		Ref		34 (7.2)		Ref		24 (5.1)		Ref	

^a^RR: risk ratio.

^b^The low-risk and at-risk groups were coded as 0, and the high-risk group was coded as 1.

^c^No reference group.

^d^N/A: not applicable (cannot be computed).

^e^Ref: reference group.

### Sensitivity and Specificity Analysis

The sensitivity and specificity analyses are summarized in Table 4. The various classification schemes generally had a high sensitivity (83%-100%) for both the UK and the US samples, with the exception of the polythetic scheme that had somewhat lower sensitivity (74% and 73% for the UK and the US samples, respectively). The specificity and negative predictive value were also high across the various schemes (>88% and >95%). Moreover, there were considerable consistencies or overlaps (>88%) in group membership between the latent profiles and the high-versus-low-risk groups classified by all the schemes, indicating that the participants classified by the various schemes were largely consistent with the latent groups embedded in the data when their sensitivity, specificity, and negative predictive value were examined.

**Table 4:** Sensitivity and specificity analyses of various classification schemes with the latent group as the benchmark (N=1047).

Sample and classification scheme			Indicator of sensitivity and specificity analyses (%)								
			Sensitivity		Specificity		Positive predictive value		Negative predictive value		Overall consistency
**The UK sample (n=573)**											
	Strict monothetic	100		89		6		100		89	
	Strict polythetic	100		92		34		100		92	
	Monothetic	100		93		43		100		93	
	Polythetic	74		99		94		96		95	
**The US sample (n=474)**											
	Strict monothetic	100		91		2		100		91	
	Strict polythetic	83		93		23		100		93	
	Monothetic	100		93		28		100		93	
	Polythetic	73		99		88		97		96	

Despite these similarities among the schemes, it is important to note that the schemes differed vastly in their positive predictive value. Only the polythetic scheme had a high positive predictive value for both samples (>88%), whereas the other schemes had a low positive predictive value (<43%). These results indicated that only the polythetic scheme could identify high-risk participants that were largely consistent with the data, whereas all other schemes might fail to identify a considerable proportion of participants who were classified as high risk by the data-driven LPA.

In summary, the various classification schemes generally had good sensitivity and specificity, but their positive predictive value was low, indicating that those participants who were classified by these schemes as having high risks only represented a relatively small proportion of participants from the data-driven, latent high-risk group. Despite having somewhat lower sensitivity than other schemes, the polythetic scheme had a high positive predictive value, indicating that the membership of various groups derived from the polythetic scheme overlapped with the group membership identified by the data-driven latent profiles as much as 96% of the time. Taken together, the polythetic scheme yielded the best balance of sensitivity, specificity, positive predictive value, and negative predictive value among the major classification schemes, and may thus be optimal for classifying cases of social media addiction.

### Risk Ratio of Mental Health Problems by Classification Scheme and Criteria

Table 5 presents the descriptive statistics of mental health problems for both low-to-at-risk and high-risk groups classified by various schemes, whereas Table 6 displays the risk ratios of various major mental health problems associated with the incidence of social media addiction at both the scale and item levels. In these analyses, the reference group referred to the low-to-at-risk group classified by various schemes of social media addiction.

**Table 5:** Descriptive statistics of mental health problems by case classification of major schemes for the 2 samples (N=1047).

Scheme and mental health problem			The UK sample (n=573)							The US sample (n=474)				
			High-risk group, mean (SD)		Low-to-at-risk group, mean (SD)		*P* value^a^		High-risk group, mean (SD)			Low-to-at-risk group, mean (SD)		*P* value
**Strict monothetic**														
	Depression	41.25 (9.64)		16.87 (11.08)		<.001		N/A^b^			N/A		N/A	
	Anxiety	64.25 (12.61)		40.07 (13.11)		<.001		N/A			N/A		N/A	
**Strict polythetic**														
	Depression	32.39 (11.26)		16.40 (10.79)		<.001		26.08 (8.62)			16.11 (10.89)		.002	
	Anxiety	56.13 (13.88)		39.56 (12.80)		<.001		48.25 (14.52)			38.39 (13.12)		.01	
**Monothetic**														
	Depression	27.03 (13.30)		16.51 (10.88)		<.001		26.00 (11.08)			16.12 (10.83)		.002	
	Anxiety	52.90 (14.20)		39.55 (12.86)		<.001		52.00 (11.95)			38.32 (13.11)		.001	
**Polythetic**														
	Depression	26.02 (12.67)		15.47 (10.21)		<.001		24.65 (10.38)			15.36 (10.58)		<.001	
	Anxiety	48.35 (13.94)		38.76 (12.59)		<.001		45.25 (13.09)			37.83 (13.04)		<.001	
**Latent profile^c^**														
	Depression	26.59 (12.70)		15.75 (10.39)		<.001		24.00 (11.02)			15.62 (10.65)		<.001	
	Anxiety	50.10 (14.01)		38.87 (12.56)		<.001		45.98 (13.07)			37.91 (13.04)		<.001	

^a^*P* value indicates the significance level of an independent sample *t* test (1-tailed) for each mental health problem of a sample.

^b^N/A: not applicable (cannot be computed).

^c^The low-risk and at-risk groups were coded as 0, and the high-risk group was coded as 1.

**Table 6:** Risk ratio of mental health problems by classification scheme and criteria for social media addiction (N=1047).

Parameters			Mental health problems in the UK sample (n=573), RR^a^					Mental health problems in the US sample (n=474), RR		
			Probable depression		Probable anxiety		Probable depression			Probable anxiety
**Classification scheme^b^**										
	Strict monothetic	N/A^c^		N/A		N/A			N/A	
	Strict polythetic	5.99		6.17		6.59			14.81	
	Monothetic	2.77		8.48		3.92			2.90	
	Polythetic	4.46		3.27		6.21			2.65	
	Latent profile	4.17		3.85		4.60			2.79	
**Criteria^d^**										
	Salience	1.97		2.18		2.51			1.68	
	Tolerance	2.58		2.16		3.35			2.08	
	Mood modification	4.34		3.54		6.33			2.99	
	Loss of control	1.57		1.38		2.62			2.29	
	Withdrawal	5.99		3.52		7.29			8.95	
	Conflict	7.02		2.79		3.88			4.33	
**Criteria^e^**										
	Salience	1.44		1.67		2.19			1.66	
	Tolerance	1.29		1.63		2.82			1.81	
	Mood modification	3.93		2.83		5.22			3.20	
	Loss of control	2.37		1.87		3.06			3.09	
	Withdrawal	3.17		3.54		4.60			2.79	
	Conflict	4.08		3.79		3.30			2.65	

^a^RR: risk ratio, with the low-risk and at-risk groups coded as 0, and the high-risk group was coded as 1.

^b^This analysis was conducted at the scale level.

^c^N/A: not applicable (cannot be computed).

^d^This analysis was conducted at the item level with a cutoff score of 4.

^e^This analysis was conducted at the item level with a cutoff score of 3.

For the UK sample, the risk of probable depression or anxiety was about 3 to 8 times higher in the high-risk group by various schemes than in the low-to-at-risk group. Similarly, for the US sample, the risk of having any of these mental health problems was about 3 to 15 times higher for the high-risk (vs no-to-low-risk) group identified by various schemes.

As the profiles of the latent groups revealed some interesting patterns across the 6 criteria for social media addiction, additional analyses were conducted at the item (criterion) level. Specifically, each of the BSMAS items was dummy coded according to the cutoff adopted in the strict monothetic and strict polythetic schemes (ie, 4 out of a 5-point scale). As shown in the middle panel of Table 6, the risk of probable depression or anxiety was about 3 to 9 times higher for the mood modification, withdrawal, and conflict criteria for both the UK and the US samples when a high cutoff of 4 was applied.

The BSMAS items were also dummy coded according to the cutoff adopted in the monothetic and the polythetic schemes (ie, 3 out of a 5-point scale), and the results are summarized in the lower panel of Table 6. Similar to the findings derived from a higher cutoff of 4, the risk of probable depression or anxiety was about 3 to 5 times higher for the mood modification, withdrawal, and conflict criteria for both the UK and the US samples.

## Discussion

### Principal Findings

Social media addiction has emerged as a prevalent problem of public concern in the modern cyber era, and this emergent problem has been examined in the context of the COVID-19 pandemic. Although the prevalence rates of social media addiction for both samples obtained in this period are comparable with those derived from the same countries before the pandemic [61], the psychiatric problems reported by both samples are more prevalent than those reported in previous studies [62]. These findings indicate that the residents of the United Kingdom and the United States could be emotionally overwhelmed by enormous stressors elicited during the early phase of the pandemic [62,63], but the prevalence of social media addiction remained largely stable at that stage.

This study is the first to adopt a nuanced analysis of the various schemes that have been widely used for case classification purposes. Our findings indicate that the application of diverse schemes yields varied prevalence estimates, and testing these schemes against the data-driven, latent profile analytic approach thus generates valuable information that unveils the relative performance of different schemes in case classification.

In our study, three latent profiles are found to be embedded in the data: low-risk, at-risk, and high-risk. It is noteworthy that the shape of the symptom profile of the at-risk group was distinct from that of the other 2 groups. For the at-risk group, the endorsement of half of the criteria (ie, salience, tolerance, and mood modification) tended to be more similar to the endorsement of those by the high-risk group, whereas the endorsement of the other half (ie, loss of control, withdrawal, and conflict) by the at-risk group tended to be more similar to the endorsement of those by the low-risk group. These findings show that a dichotomous (low-risk vs high-risk) classification scheme may be inadequate. Instead, a tripartite classification scheme may be more appropriate for capturing respondents’ distinct symptom characteristics of social media addiction, especially for the at-risk group whose symptom profile is similar to the low-risk and high-risk groups in certain criteria but not others.

In general, most classification schemes used in existing studies have good sensitivity and specificity when compared with the latent groups identified in our latent profile analyses. However, a low positive predictive value is found in most of the schemes, with the exception of the polythetic scheme. The present findings indicate that both strict monothetic and monothetic schemes have perfect sensitivity (100%) but at the cost of low positive predictive values (2%-43%). These 2 schemes may be too conservative, such that a large proportion of individuals with probable social media addiction are excluded from further assessment or follow-ups. In contrast, despite having slightly lower sensitivity than the other schemes, the polythetic scheme appears to have a more balanced performance across all psychometric indicators.

### Research and Practical Implications

These new findings have research and practical implications. Specifically, the study delineates the strengths and weaknesses of each classification scheme, thus guiding decisions for selecting an optimal scheme for making population-level estimates of social media addiction. The findings indicate that the various major classification schemes have a large degree of heterogeneity, both in terms of estimating the prevalence rates of social media addiction as well as screening and detecting cases of social media addiction. With regard to prevalence estimates, researchers utilizing strict classification schemes may tend to obtain very low prevalence rates of social media addiction. As shown in this study, such discrepancies can be as large as 10% to 15% (eg, using polythetic vs strict monothetic schemes). It is noteworthy that the polythetic scheme was found to have relatively high consistency with the benchmark data of the 2 independent samples, with discrepancies of only 2% to 3% (11% vs 9% in the United States; 15% vs 12% in the United Kingdom). Thus, the present findings provide some empirical evidence that polythetic classification may best reflect classifications identified using a data-driven approach and is optimal when the goal is to identify a broad group of individuals at risk for social media addiction.

Furthermore, the assessment of social media addiction has become an issue of growing importance for mental health professionals, with some countries formulating public health policies and providing extensive training to guide professionals in distinguishing clients with varying severity levels of the problem [64]. As precise case classification is crucial for appropriate referral to tailored intervention, a better understanding of how well a screening instrument function using different scoring methods becomes important. In evaluating screening tools, the sensitivity and specificity of classification schemes have traditionally been emphasized. However, basing classification schemes solely on these 2 indices is insufficient for making clinical decisions at the individual level and may even be misleading in some cases [65]. This is because both indicators of sensitivity and specificity are population-based indices that represent properties of the screening test in itself [66], but the 2 predictive values further include sample-relevant information, such as the base rate of a given problem area [67]. Therefore, the use of multiple indices, including positive and negative predictive values, is a notable strength of our analyses, providing valuable data that could inform the scoring and interpretation of the BSMAS within the context of clinical assessment [65,68].

The BSMAS is a brief measure that has been commonly adopted to assess symptoms of social media addiction, although our results highlight several considerations relevant to clinicians’ choice of classification schemes. Despite demonstrating high sensitivity and specificity, the use of the strict monothetic, strict polythetic, and monothetic schemes are found to be more prone to missing members of the high-risk group derived from the actual data (ie, positive predictive values ranging from 2% to 43%) as compared with the polythetic scheme (ie, positive predictive values of 88% and 94%). These findings demonstrate that the polythetic scheme has superior positive predictive rates of social media addiction compared with the other schemes, indicating the greater utility of the polythetic scheme in detecting individuals who are at high risk for social media addiction.

The polythetic scheme as compared with the monothetic scheme is more consistent with the current theoretical approaches to psychological functioning and mental health, as the polythetic scheme emphasizes a prototypical perspective (ie, requiring the presence of many rather than all symptoms) rather than a classical, monothetic perspective (ie, requiring the presence of all symptoms) to mental health [69]. Characterized by a prototypical approach, the polythetic scheme does not necessitate the presence of all symptoms within a problem area, representing an approach that is more attuned toward individual variability and heterogeneity, both of which are frequently observed in clinical and research settings. Similar to many other problem domains, the present findings indicate that social media addiction may be conceptualized as a continuum ranging from no to high risks, with a considerable proportion of at-risk individuals clustered somewhere between the 2 extremes. Hence, the polythetic classification scheme is more similar to current assessment approaches to mental health problems, such as the DSM-5 and the 11th version of the International Statistical Classification of Diseases and Related Health Problems (ICD-11) [27,70]

Although information technology addiction (eg, social media addiction and gaming disorder) is currently not a diagnosable condition in the DSM-5 [71], a recent expert review has outlined meta-level criteria for determining whether different behavioral addictions may warrant the designation of *other specified disorders due to addictive behaviors* under the ICD-11 [72]. To determine whether social media addiction is present based on specific symptoms, this study fills the knowledge gap by revealing 3 symptoms of social media addiction with high-risk ratios with comorbid problems of depression and anxiety: mood modification, withdrawal, and conflict (Table 6). The replicable findings across the 2 independent samples suggest that these symptoms are crucial for the assessment of social media addiction. More importantly, these findings echo the classifications of a related problem of gaming disorder and other behavioral disorders in the ICD-11, in which conflict and adverse consequences in significant life domains are essential for a diagnosis.

This study further reveals that individuals identified with social media addiction have a high risk of probable depression and anxiety. Primarily, these findings indicate that the classification schemes of social media addiction may serve as a risk indicator in the screening process for potentially detecting comorbid problems with depression and anxiety. More importantly, several specific symptoms of social media addiction tend to have stronger connections with depression and anxiety, including mood modification, withdrawal, and conflict. Our item-level analyses identified several associations that warrant future research and attention by clinicians. First, the association between the specific symptoms of conflict and depression and anxiety corroborates those that have been empirically unveiled in a variety of contexts [73,74], highlighting conflict arising from social media addiction as a potential pathway linked with both mental health conditions. Clinicians may need to evaluate how conflict arising from social media addiction is related to depressive symptoms, such as the feelings of letting oneself and their significant others down, feelings of worthlessness, or anxiety symptoms associated with the monitoring of worry and rumination thoughts that are attributable to addictive use of social media [75,76]. Second, mood modification through addictive use of social media to avoid facing real-life problems is associated with a high risk of probable depression and anxiety. This finding may reflect individuals who use social media as a refuge to evade real-life challenges, duties, or responsibilities. If this is the case, clinicians may need to address social media addiction as a maladaptive, avoidant coping response that likely serves as a maintaining factor for both depression and anxiety [77,78]. Finally, as withdrawal symptoms are associated with psychological distress, individuals who are at risk for social media addiction may benefit from developing alternative coping skills that can replace social media activities when experiencing withdrawal.

In addition to the use of the BSMAS as a diagnostic tool for identifying individuals with social media addiction for treatment referral, this screening tool can also serve as an effective tool for early screening of at-risk cases to prevent further development into social media addiction. As prevention is often more cost-effective than treatment [79], broad-based screening can be valuable when mental health intervention resources are available in the community to serve the identified at-risk individuals. Given that this study evaluates the performance of multiple classification schemes against the empirically-derived benchmark, the findings can provide useful guidance for health care professionals to select the scheme most appropriate for their intervention schemes.

### Research Limitations and Directions

Before concluding, caution should be exercised. First, this study adopted a quantitative design that included only validated measures with structured close-ended questions. The present inquiry thus focused on the typical symptoms of social media addiction, anxiety, and depression. The widely adopted quantitative design should be supplemented with qualitative data collection methods, such as narrative interviews and focus groups, which can broaden the scope of inquiry by unveiling participants’ unique experiences [80,81]. Thus, a mixed methods design is encouraged to combine quantitative and qualitative methods to gain a more comprehensive perspective on social media addiction.

Second, our study used a web-based survey method for data collection and is thus vulnerable to the shortcomings inherent in this type of method. Although the screening function of the web-based survey platform allows the recruitment of the present samples whose demographic profiles resemble those of their respective populations, it is important to reiterate that nonprobabilistic sampling method was used in participant recruitment. Participants signed up for the web-based survey voluntarily upon placing an advertisement on the website, and such self-selection could potentially elicit sample bias.

Third, our study adopted a symptom approach in the examination of social media addiction and its mental health implications. Validated measures assessing a standard set of symptoms of social media addiction and the 2 psychiatric problems were administered. It is noteworthy that we did not include any measures of time for overall internet use or social media engagement [82,83]; thus, the amount and pattern of social media use were not assessed. For criterion assessment, only anxiety and depression were measured because both are major psychiatric comorbidities of information technology addiction [84,85]. Apart from psychiatric problems, individuals with information technology addiction also experience disruptions in other life domains, such as interpersonal relations and job performance [86,87]. The scope of daily life dysfunction should be broadened by including a greater variety of life domains for a more comprehensive evaluation of daily life challenges experienced by individuals with social media addiction.

Fourth, it is noteworthy that this study was conducted during the COVID-19 pandemic. As the massive global transmission of this unknown virus was unprecedented, an avalanche of false and misleading information was disseminated through social media. Frequent use of social media was associated with COVID-19 anxiety that disturbed sleep quality [88,89]. Such psychological responses may not be fully captured by the traditional measures of mental health. Future research may consider using measures that capture COVID-19–specific stressors and experiences, such as loss of family and friends because of COVID-19 or exposure to misinformation about the pandemic via social media use [90].

Finally, although efforts have been made to replicate the findings in 2 independent samples from English-speaking countries with high internet penetration rates [50], the present findings cannot be generalized to individuals from other countries or cultural backgrounds. This is particularly the case as multinational meta-analyses have revealed the prevalence of information technology addiction and its differential underlying psychological mechanisms across cultural regions [61,84]. Therefore, future research should be expanded to include more countries with varying levels of cultural individualism and internet penetration. Data derived from a myriad of countries with diverse backgrounds enable researchers to make cross-cultural comparisons at both the individual and country levels [91].

### Conclusions

In conclusion, this study evaluated 4 major schemes widely adopted to classify cases of social media addiction. Using latent profiles identified from empirical data as a benchmark, the performance of the polythetic scheme is more well-balanced in attaining relatively high levels of sensitivity, specificity, positive predictive value, and negative predictive value compared with those of the other 3 schemes. Although these findings are largely replicable in the 2 independent samples, efforts should be made to expand the scope of inquiry in countries with diverse cultural backgrounds using a wider range of criterion variables through qualitative methods, thus enriching the discussion and informing future decisions about the potential inclusion of social media addiction in the future versions of the DSM or ICD.

## Abbreviations

BSMAS: Bergen Social Media Addiction Scale
DSM: Diagnostic and Statistical Manual of Mental Disorders
ICD: International Statistical Classification of Diseases and Related Health Problems
LPA: latent profile analysis
